# Quality improvement supervision comparison between training and non training posts

**DOI:** 10.1192/bjo.2021.527

**Published:** 2021-06-18

**Authors:** Qutub Jamali, Tarun Khanna, Gareth Thomas

**Affiliations:** Lancashire Care NHS Foundation Trust

## Abstract

**Aims:**

To explore the level of supervision between training and non-training posts at LSCFT.

**Background:**

- Supervision is defined as ‘provision of guidance and feedback on matters of personal, professional and educational development in the context of a trainees' experience of providing safe and appropriate patient care’.
- Along with the trainees, doctors working in non-training posts such as staff grade, specialty doctors, trust grade doctors (TJD)and MTI (Medical training initiative) doctors form an integral part of patient care in the NHS.

**Method:**

- A mixed method approach was adopted with both qualitative and quantitative data collected simultaneously in the form of an online questionnaire.
- An anonymous online questionnaire was sent to junior doctors currently in training and non-training posts at LSCFT in 2019 using Meridian software.

**Result:**

1- Quantitative Data: - Participants included were doctors in training post such as Foundation Doctors (5), Psychiatry Core Trainees (6), GP STs (2) and doctors in non-training post such as TJD (4), Specialty Doctors (2) and MTI doctors (4). Based on the Meridian score, 84% of doctors were satisfied with the supervision. It was found that 72% of doctors received weekly supervisions, 10% monthly (1 TJD, 1 Foundation trainee) and16% bi-monthly (1 MTI, 1 SAS, 2 CTs). The data suggested that there was no difference in the frequency of supervisions between training and non-training posts at LSCFT.

2- Qualitative Data: - The feedback was common as there was no major difference between training and non-training doctors.

- Positives – WPBAs, discussion on reflections, management of complex cases and medication, personal issues affecting work.
- Negatives – Limited discussion on QI, Audit, Research and Psychotherapy.

- More specific help, need more support at times.

**Conclusion:**

1. To prepare a checklist of contents to be discussed during supervision.
2. To prepare a timeline chart of supervision.
3. Preparing a ‘menu’ of QI projects that junior doctors can sign up to at the start of each post.
4. To formulate training packages available to support junior doctors with QI/Audits.

